# Supplementary material for: Syndapin-2 mediated transcytosis of amyloid-β across the blood–brain barrier
Source: Brain Commun. 2022 Feb 17;4(1):fcac039. doi: 10.1093/braincomms/fcac039 (PMC8882007; doi:10.1093/braincomms/fcac039)
Supplement: fcac039_Supplementary_Data [file fcac039_supplementary_data.docx]

**Supplementary Materials**

**Syndapin-2 mediated transcytosis of amyloid-ß across the blood-brain barrier**

Diana M. Leite^1,2 *^, Mohsen Seifi^3^, Lorena Ruiz-Perez^1,2^, Filomain Nguemo^4^, Markus Plomann^5^, Jerome D. Swinny^6^, Giuseppe Battaglia ^1,2,7,8 *^

^1^ Department of Chemistry, University College London, London, United Kingdom.

^2^ Institute for the Physics of Living Systems, University College London, London, United Kingdom.

^3^ Leicester School of Pharmacy, Faculty of Health and Life Sciences, De Montfort University, Leicester, United Kingdom.

^4^ Institute for Neurophysiology, Faculty of Medicine, University of Cologne, Cologne, Germany.

^5^ Institute of Biochemistry, Faculty of Medicine, University of Cologne, Cologne, Germany.

^6^ School of Pharmacy and Biomedical Sciences, University of Portsmouth, United Kingdom.

^7^ Institute for Bioengineering of Catalonia (IBEC), The Barcelona Institute for Science and Technology (BIST), Barcelona, Spain.

^8^ Catalan Institution for Research and Advanced Studies (ICREA), Barcelona, Spain.

**Table 1.** List of antibodies.

| **Antibody** | **Dilution** | **Supplier, Catalogue Number** |
| --- | --- | --- |
| Rabbit polyclonal to syndapin-2 | 1:400 | Abcam, ab37615 |
| Mouse monoclonal to syndapin-2 | sc-390136 | Santa Cruz Biotechnology, Sc-390136 |
| Mouse monoclonal to LRP1 | 1:100 | Sigma, L2420 |
| Mouse monoclonal to LRP1 | 1:1000 | Invitrogen, 37-7600 |
| Rabbit monoclonal to LRP1 | 1:1000 | Abcam, ab92544 |
| Mouse monoclonal to GAPDH | 1:1000 | Abcam, ab8245 |
| Mouse monoclonal to ApoE | 1:200 | Novus Biologicals, NB110-60531 |
| Mouse monoclonal to RAGE | 1:100 | Santa Cruz Biotechnology, sc-365154 |
| Mouse monoclonal to p-glycoprotein | 1:100 | Invitrogen, MA1-26528 |
| Mouse monoclonal to Clathrin Heavy Chain | 1:1000 | Abcam, ab2731 |
| Rabbit polyclonal to Clathrin Heavy Chain | 1:100 | Abcam, ab21679 |
| Rabbit polyclonal to dynamin-2 | 1:100 | Abcam, ab 3357 |
| Mouse monoclonal to EEA-1 | 1:100 | Sigma, E7659 |
| Rabbit polyclonal to EEA-1 | 1:100 | Abcam, ab2900 |
| Mouse monoclonal to Rab5 | 1:400 | Sigma, R7904 |
| Rabbit polyclonal to Rab5 | 1:100 | Abcam, ab13253 |
| Rabbit polyclonal to PICALM | 1:100 | Sigma, HPA019061 |
| Mouse monoclonal to Rab7 | 1:100 | Sigma, R8879 |
| Rabbit polyclonal to Rab7 | 1:100 | Abcam, ab137029 |
| Rabbit polyclonal to Rab11 | 1:100 | Abcam, ab3612 |
| Rabbit polyclonal to LAMP-1 | 1:100 | Abcam, ab24170 |
| Rabbit polyclonal to ß-actin | 1:100 | Abcam, ab8227 |
| Mouse monoclonal to A𝛽 | 1:1000 | Biolegend, 800708 |
| Rabbit polyclonal to claudin-5 | 1:1000 | Abcam, ab15106 |
| Rabbit polyclonal to occludin | 1:1000 | Abcam, ab216327 |
| Alexa Fluor 488 goat anti-mouse IgG | 1:500 | Biolegend, 405319 |
| Alexa Fluor 647 donkey anti-rabbit IgG | 1:500 | Biolegend, 406414 |
| Dylight 800 goat anti-mouse IgG | 1:5000 | Thermo Fisher Scientific, SA535521 |
| Dylight 800 goat anti-rabbit IgG | 1:5000 | Thermo Fisher Scientific, SA535571 |

**
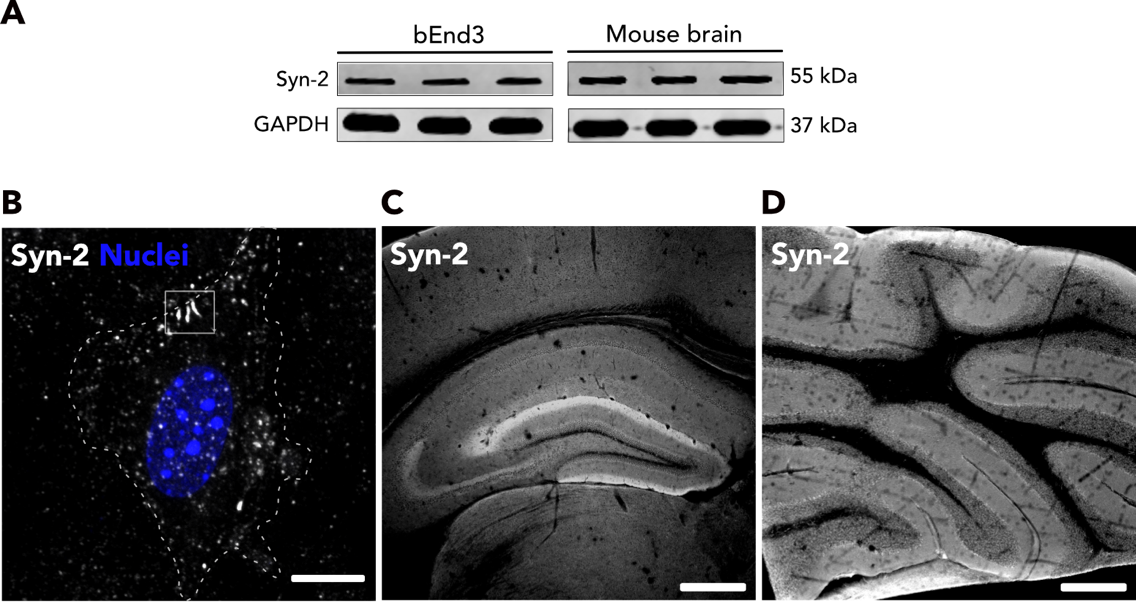
**

**Supplementary Figure 1. Expression of syndapin-2 in the brain endothelium and mouse brain.**

(**A**) Immunoblotting for syndapin-2 and GAPDH (loading control) in polarised BECs (bEnd3) and wild-type mouse brains. (**B**) Expression of syndapin-2 (in white) in BECs, highlighting syndapin-2-tubular structures. Nucleus is shown in blue and dotted line represents the cell membrane limits. Scale bar: 20 µm. Immunocolocalisation of syndapin-2 in (**C**) hippocampus and (**D**) cerebellum in mouse brain. Scale bar: 500 µm. For full blots see Supplementary Figure 12.


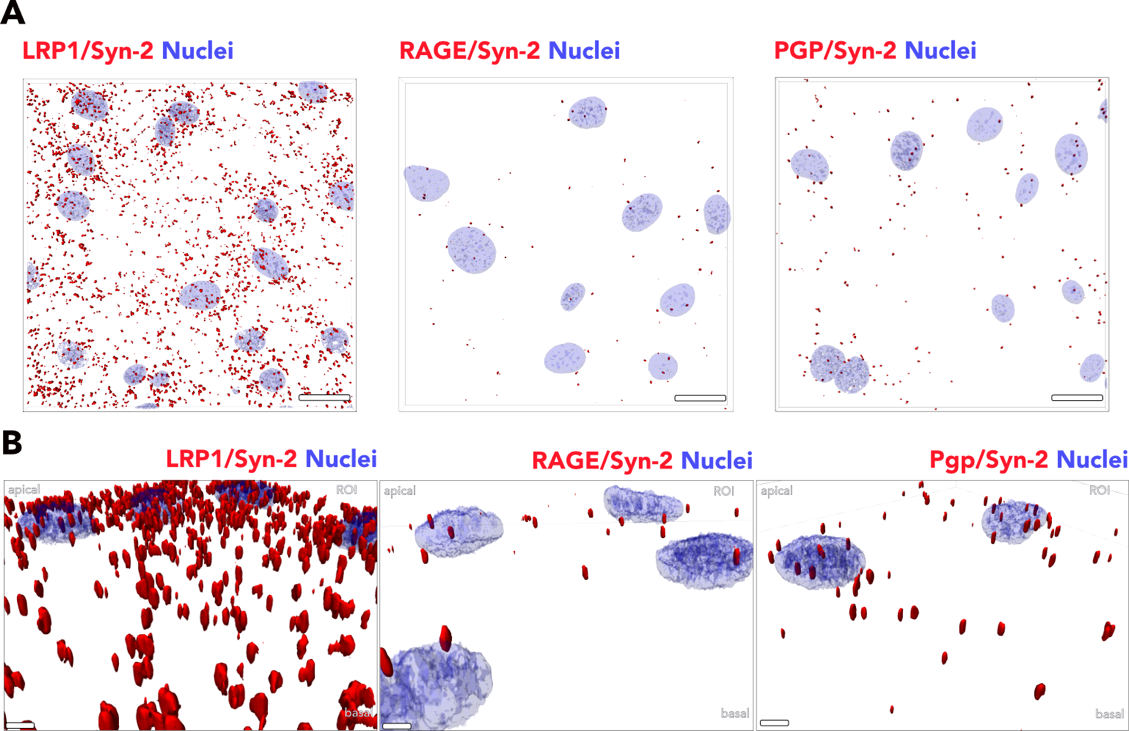


**Supplementary Figure 2. Syndapin-2 interaction with LRP1, RAGE and PGP in brain endothelium.**

(**A**) Representative confocal images of the proximity obtained for LRP1, RAGE or PGP with syndapin-2 in polarised BECs. Nuclei are shown in blue. Scale bar: 20 µm. (**B**) 3D renderings of BECs showing PLA dots between syndapin-2 and LRP1/RAGE/PGP. Scale bar: 2 µm.


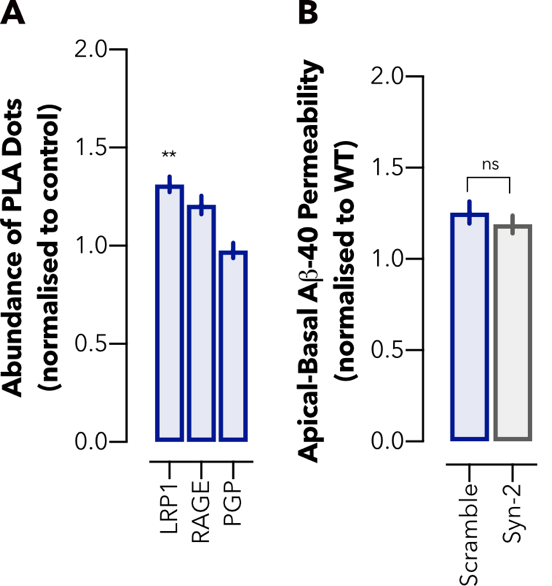


**Supplementary Figure 3. Syndapin-2 interaction with LRP1, RAGE and PGP in brain endothelium for transport of Aß.**

(**A**) Abundance of PLA dots resulting from the interaction of syndapin-2 with LRP1, RAGE or PGP in the polarised BECs treated with FAM-Aß (1-40) (500 nM) for 15 minutes. Each point represents the abundance of PLA dots in one image. Mean ± SEM (*n* = 60 images). (**B**) *In vitro* permeability of FAM-Aß (1-40) across shRNA control (scramble) and syndapin-2 knockdown (Syn-2) in an apical-to-basal (blood-to-brain) direction. Permeability values were normalised to wild-type (WT) BECs. Each data point represents the permeability of Aß across a Transwell membrane. Mean ± SD (*n* = 15). * *P* < 0.05, Student’s t-test.


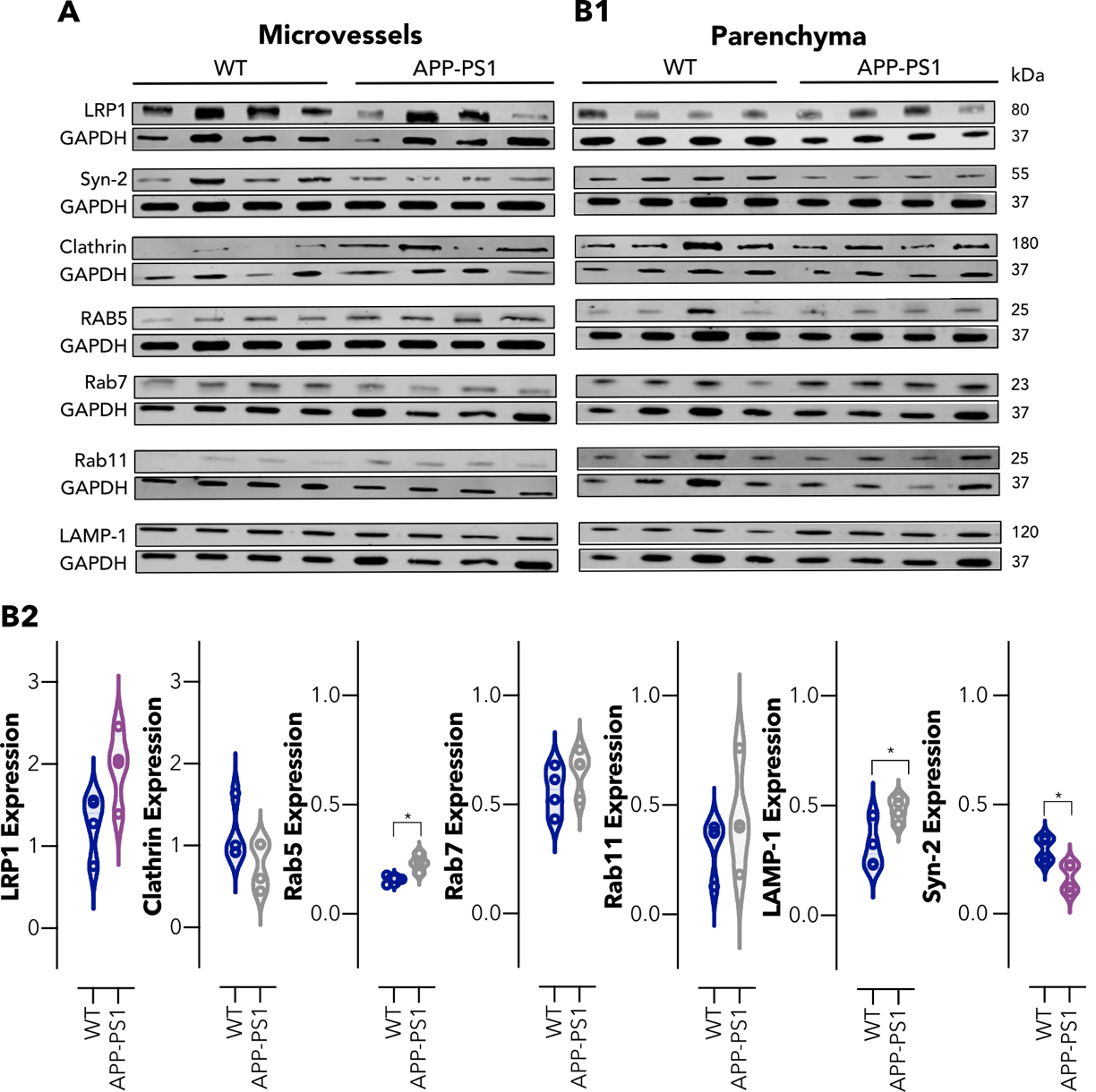


**Supplementary Figure 4. Alterations in intracellular trafficking in 12-months old WT and APP-PS1 mouse brains.** Immunoblotting for endocytic proteins in (**A**) microvessel and (**B1**) parenchymal fractions of 12-months old WT and AD (APP-PS1) mouse brains. (**B2**) Relative abundance of LRP1, clathrin, Rab5, Rab7, Rab11 LAMP-1 and syndapin-2 in the parenchyma WT and APP-PS1 mouse brains. Data normalised to loading control (GAPDH). Mean ± SD (*n* = 4 animals). * *P* < 0.05, Student’s t-test. For full blot see Supplementary Figure 13.


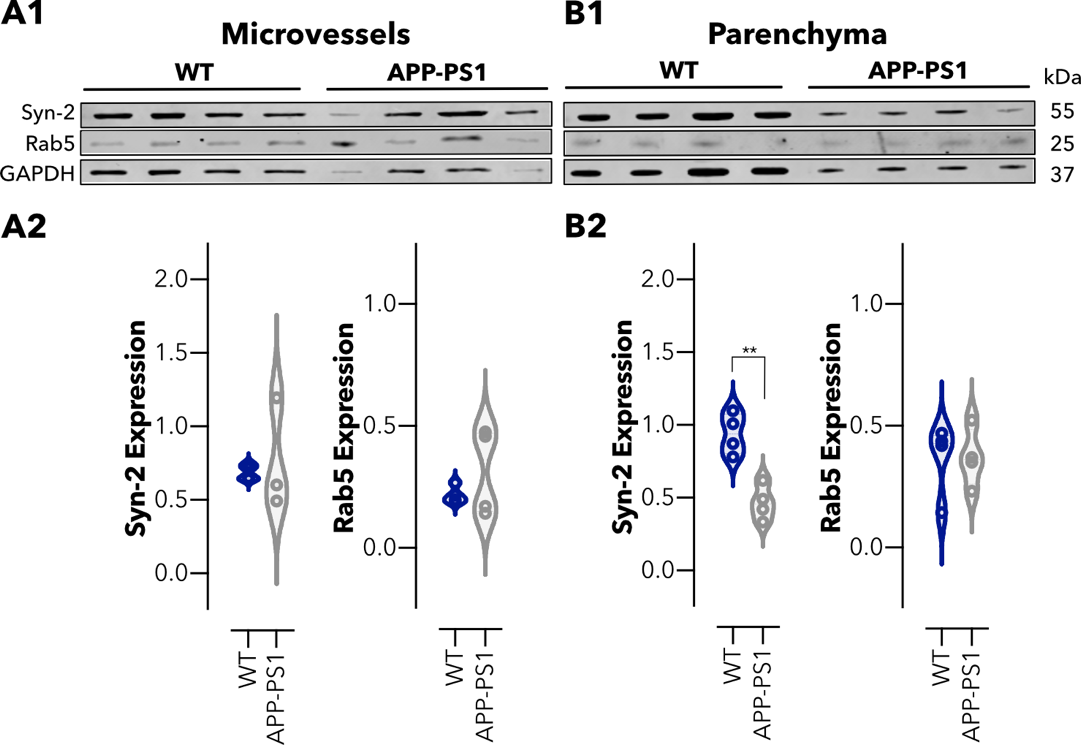


**Supplementary Figure 5. Expression of tubular and vesicular endosomal transport proteins in 4-months old WT and APP-PS1 brains.** Immunoblotting for syndapin-2, Rab5 and GAPDH (loading control) in isolated (**A1**) microvessels and (**B1**) parenchyma of 4-months old WT and APP-PS1 mouse brains. Relative abundance of syndapin-2 and Rab5 in (**A2**) microvessels and (**B2**) parenchymal fractions of WT and APP-PS1 mouse brains. Data normalised to GAPDH. Mean ± SD (*n* = 4 animals). NS, non-significant, ** *P* <0.01, Student’s t-test. For full blots see Supplementary Figure 14.


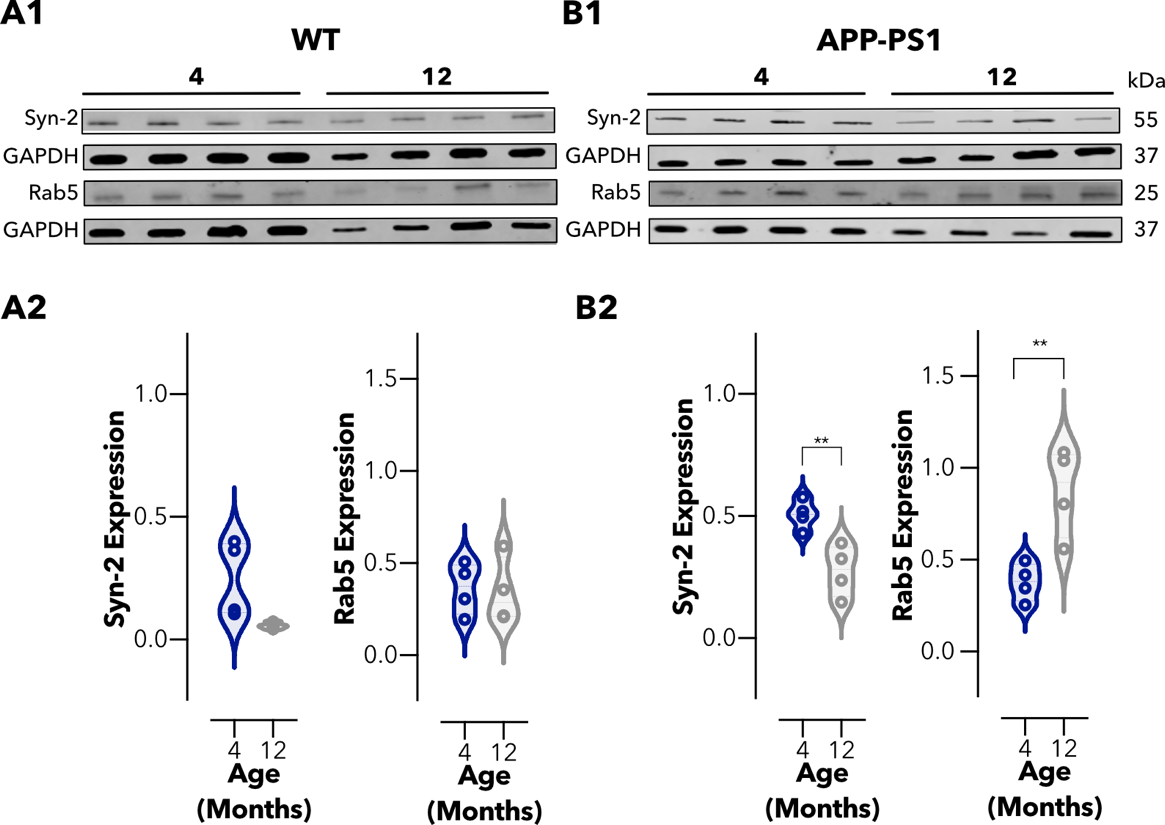


**Supplementary Figure 6. Imbalance in the level of tubular and vesicular endosomal proteins in 4- and 12-months old WT and APP-PS1 parenchyma.** Immunoblotting for syndapin-2, Rab5, and GAPDH (loading control) in the parenchyma of 4- and 12-months old (**A1**) WT and (**B**1) APP-PS1 brains. Relative abundance of syndapin-2 and Rab5 levels in the parenchyma of (**A2**) WT and (**B2**) APP-PS1 brains. Data normalised to loading control (GAPDH). Mean ± SD (*n* = 4 animals). ** *P* < 0.01, Student’s t-test. For full blots see Supplementary Figure 16.


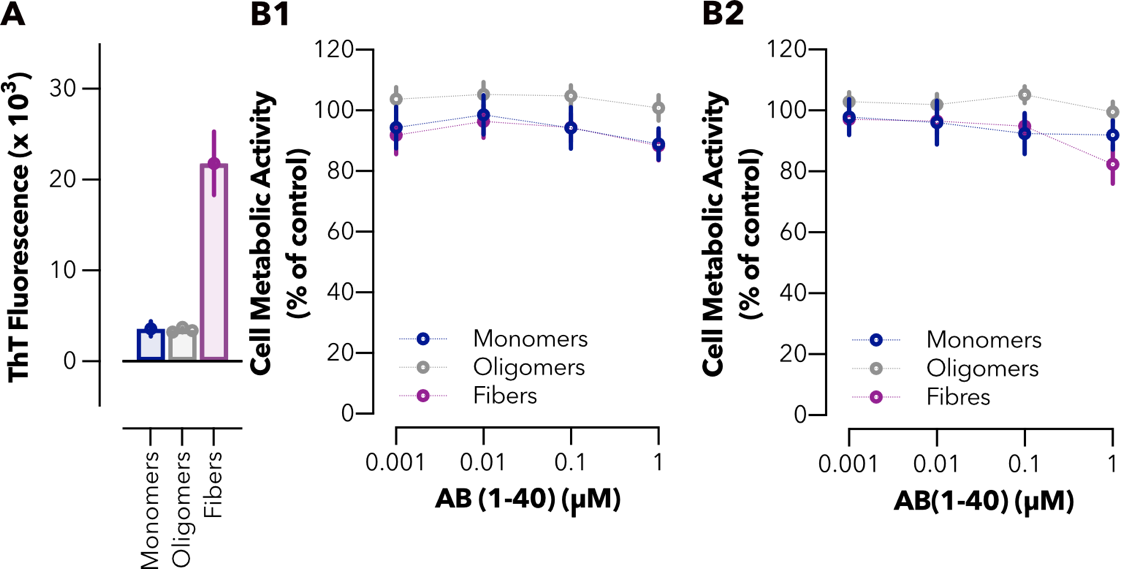


**Supplementary Figure 7. Aß characterisation and effect on brain endothelium viability.**

(**A**) Thioflavin T (ThT) fluorescence intensity after incubation with Aß monomers, oligomers, and fibrils at 10 µM. Each data point represents the fluorescence of ThT in different samples of Aß species. Mean ± SD (*n* = 3). Cell metabolic activity of BECs treated with Aß assemblies at concentrations ranging 0.001-1 µM for (**B1**) 1 and (**B2**) 24 hours. Each data point represents the mean cell viability from each cell culture replicate obtained from 4 measurements. Mean ± SD from three cell culture replicates (*n* = 3). NS, non-significant, Two-way ANOVA.

**Full Western Blots**

**
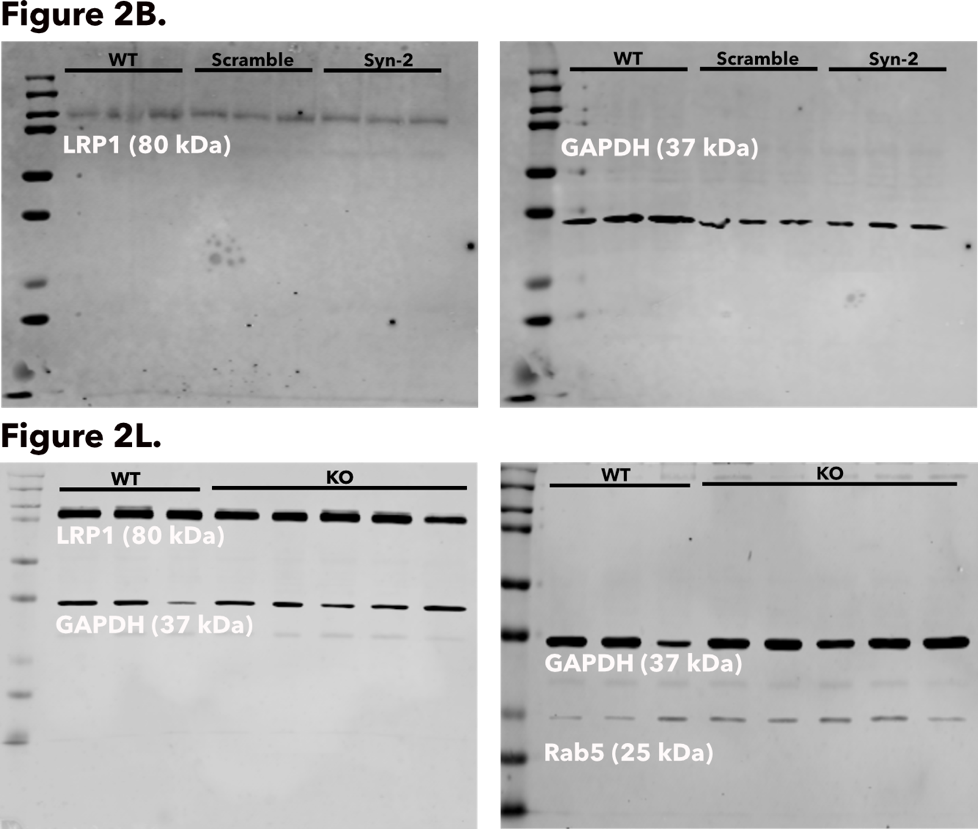
**

**Supplementary Figure 8. Full blots of the data represented in Figure 2B and 2L.**


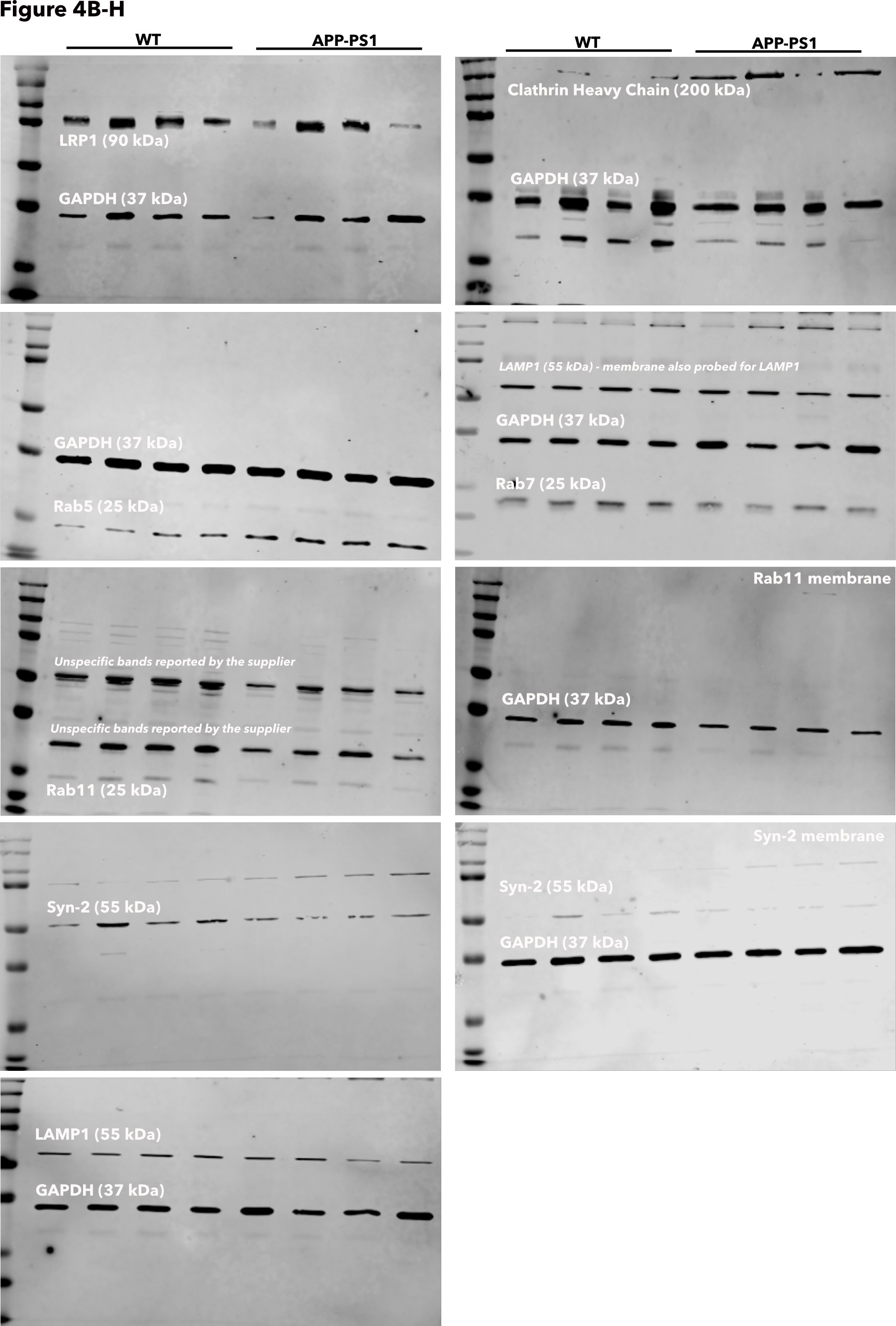
**Supplementary Figure 9. Full blots of the data represented in Figure 4B-H.
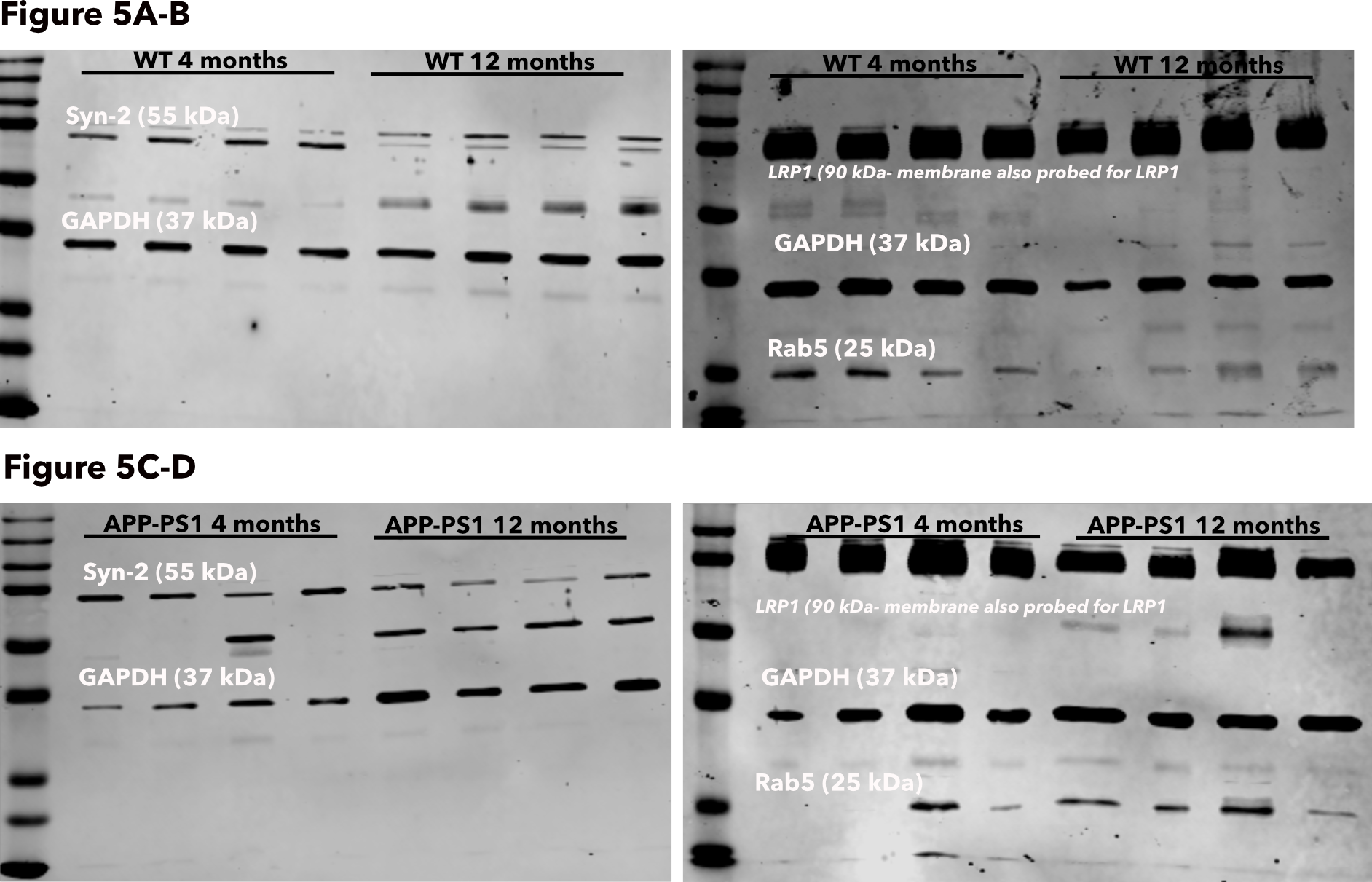
**

**Supplementary Figure 10. Full blots of the data represented in Figure 5A-B and 5C-D.**

**
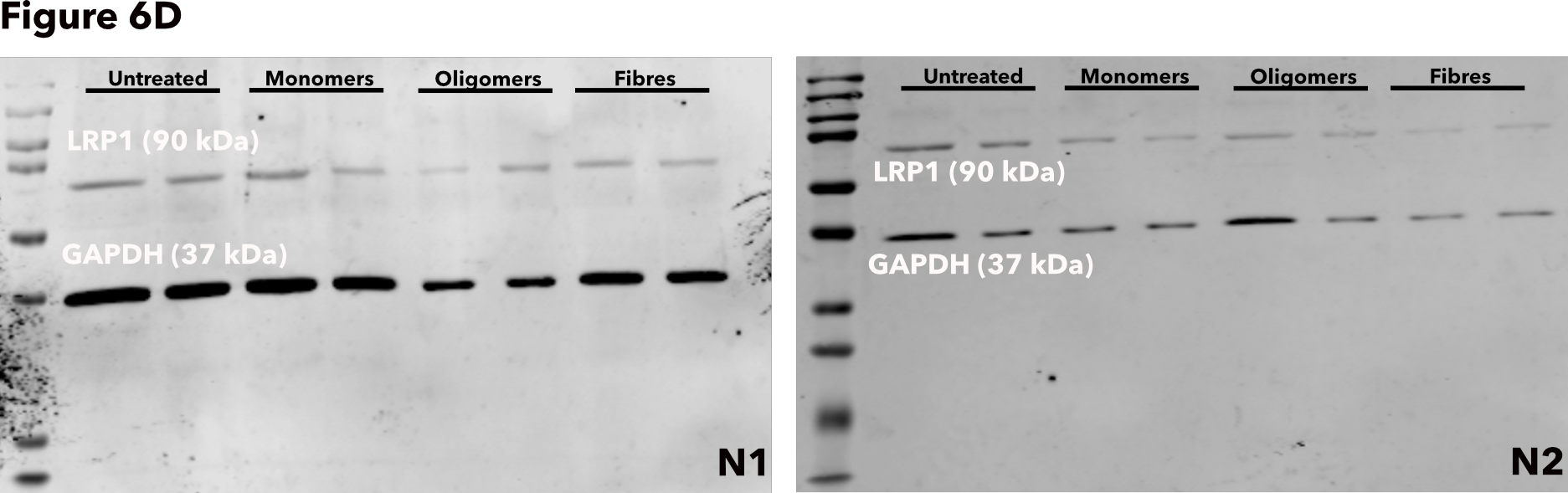
**

**Supplementary Figure 11. Full blots of the data represented in Figure 6D.**

**
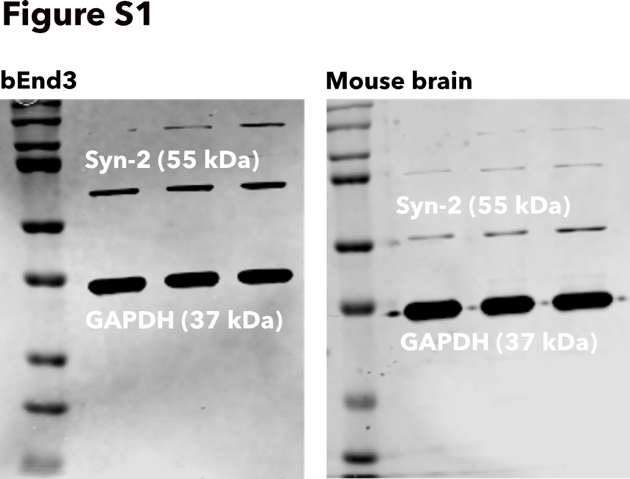
**

**Supplementary Figure 12. Full blots of the data represented in Supplementary Figure 1.**

**
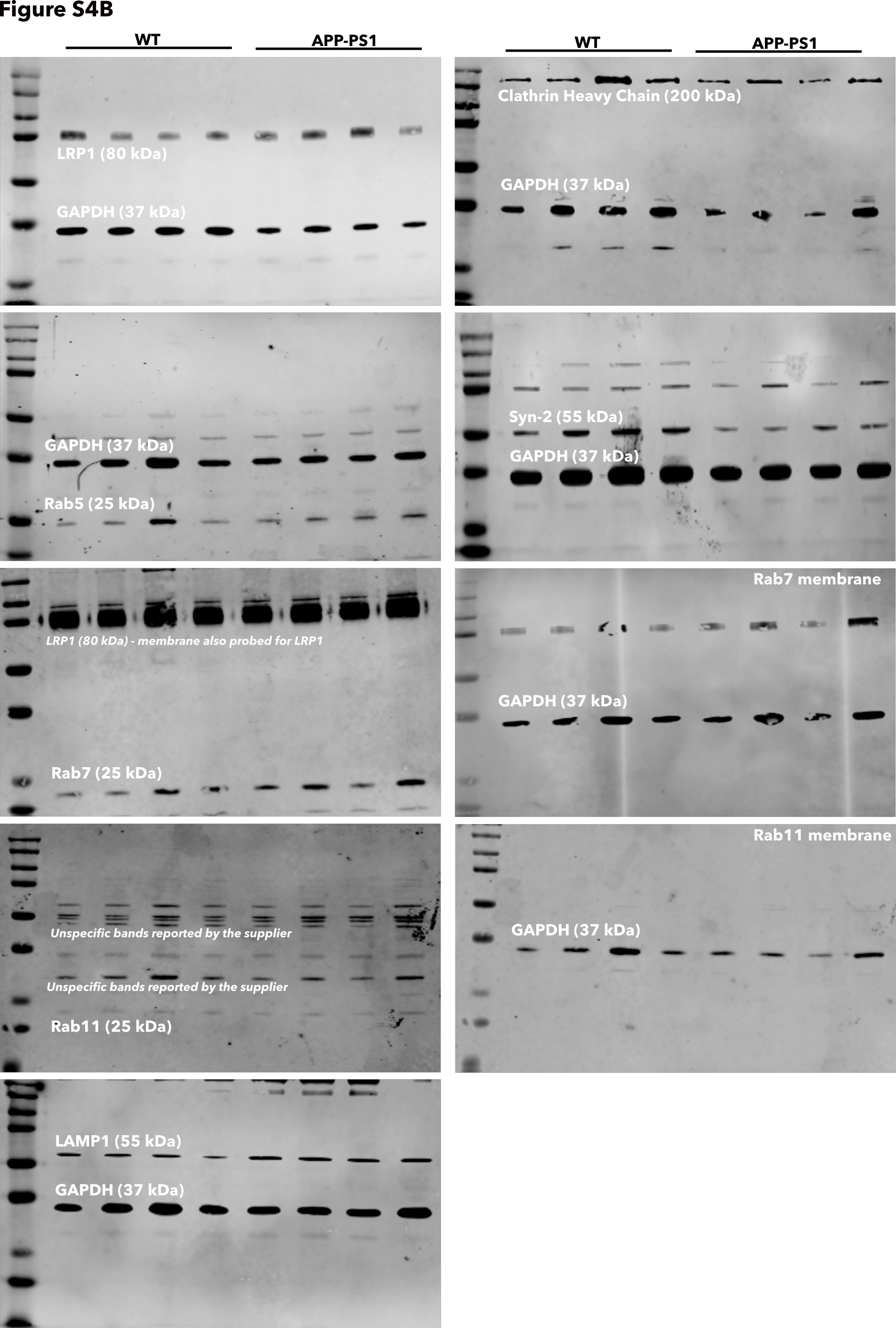
**

**Supplementary Figure 13. Full blots of the data represented in Supplementary Figure 4B.**

**
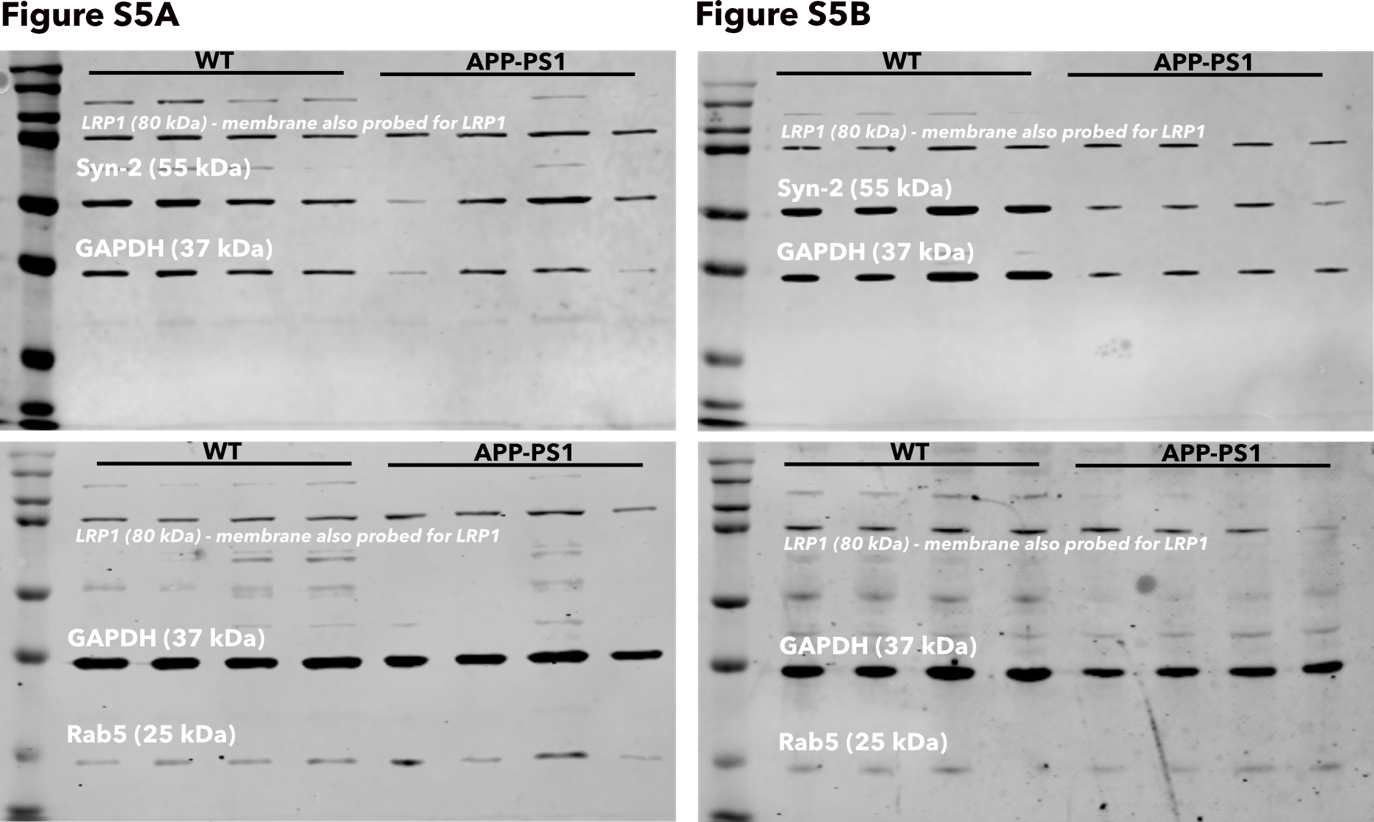
**

**Supplementary Figure 14. Full blots of the data represented in Supplementary Figure 5A (left) and 5B (right).**

**
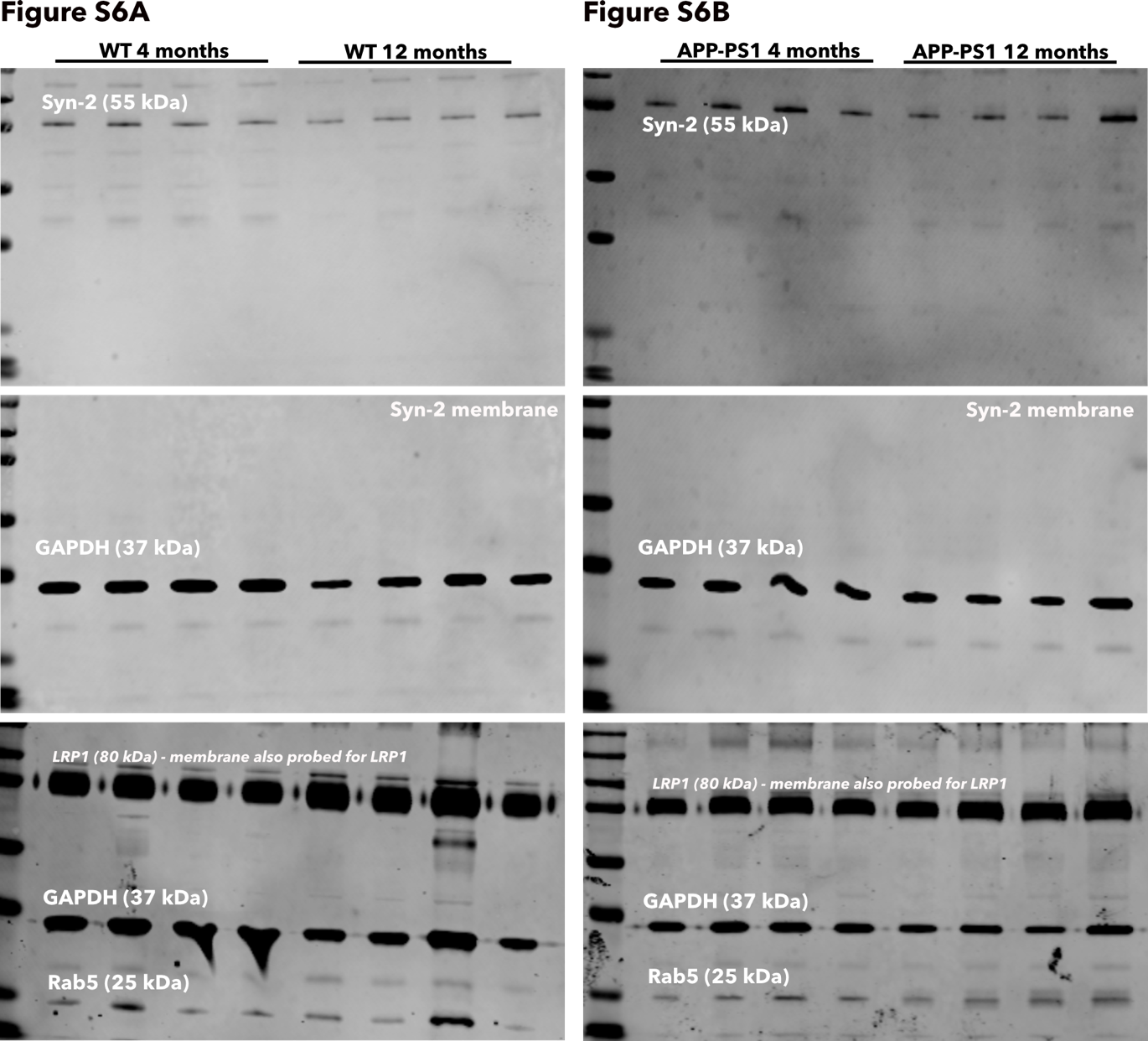
**

**Supplementary Figure 15. Full blots of the data represented in Supplementary Figure 6A.**
